# Supplementary material for: Community composition shapes microbial-specific phenotypes in a cystic fibrosis polymicrobial model system
Source: eLife. 2023 Jan 20;12:e81604. doi: 10.7554/eLife.81604 (PMC9897730; doi:10.7554/eLife.81604)
Supplement: Supplementary file 1. [file elife-81604-supp1.docx]

Supplementary File 1. Minimal Bactericidal Concentration (MBC) of *P. aeruginosa* PA14 planktonic and biofilm cells treated with tobramycin exposed to the following conditions for 24 hrs.

| **Condition** | **MBC** |
| --- | --- |
| ASM + tobramycin | 62.5 µg/mL |
| PA14 monoculture supernatant + tobramycin | 62.5 µg/mL |
| Δ*lasR* monoculture supernatant + tobramycin | 62.5 µg/mL |
| Community supernatant (WT) + tobramycin | 62.5 µg/mL |
| Community supernatant (Δ*lasR*) + tobramycin | 62.5 µg/mL |
