# Supplementary material for: Community composition shapes microbial-specific phenotypes in a cystic fibrosis polymicrobial model system
Source: eLife. 2023 Jan 20;12:e81604. doi: 10.7554/eLife.81604 (PMC9897730; doi:10.7554/eLife.81604)
Supplement: Supplementary file 2. [file elife-81604-supp2.docx]

Supplementary File 2. Strains and plasmids used in the study.

| **Species and strain** | **Strain number** | **Phenotype/Genotype** | **Ref.** |
| --- | --- | --- | --- |
| ***P. aeruginosa*** | | | |
| PA14 | SMC232 | Laboratory reference strain | (1) |
| PA14Δ*lasR* | SMC5021 | in-frame deletion of *lasR* gene | (2) |
| PA14Δ*lasR*::*lasR* | SMC9421 | SMC5021 with complementation of *lasR* at the native locus | This study |
| PA14Δ*mvfR* | SMC5018 | In-frame deletion of *mvfR* gene | (3) |
| PA14Δ*rhlR* | DH2742 | In-frame deletion of *rhlR* gene | (4) |
| PA14ΔΔ*phz* | SMC5020 | In-frame deletions of *phzA1-G1* and *phzA2-G2* genes | (5) |
| PA14Δ*mvfR*Δ*lasR* | DH1111 | In-frame deletion of *lasR* and *mvfR* genes | (3) |
| PA14Δ*lasR*Δ*rhlR* | DH2944 | In-frame deletion of *lasR* and  *rhlR* genes | (4) |
| PA14ΔΔ*phz*Δ*lasR* | SMC9422 | In-frame deletion of *phzA1-G1*, *phzA2-G2* and  *lasR* genes | This study |
| NC-AMT0101-1-2 | DH2417 | Chronic lung infection isolate with functional LasR allele, parent of NC-AMT0101-1-1 | (6) |
| NC-AMT0101-1-1 | DH2415 | Chronic lung infection isolate related to DH2417 with LasR loss-of-function (frame shift) allele | (6) |
| PA14Δ*lasI*Δ*rhlI* | DH242 | In-frame deletions of *lasI* and *rhlI* genes | (7) |
| PAO-MW1qsc102 | DH161 | PAO1 Δ*lasI*Δ*rhlI* AHL-sensing *lacZ* bioreporter for 3-oxo-C12-HSL production | (8) |
| Clinical isolate | SMC1587 | Mucoid CF isolate | (9) |
| Clinical isolate | SMC1595 | Non-mucoid CF isolate | (9) |
| Clinical isolate | SMC1596 | Non-mucoid CF isolate | (9) |
| ***S. aureus*** | | | |
| Newman | SMC1007 | Methicillin susceptible *Staphylococcus aureus* | (10) |
| JE2 | SMC8668 | Methicillin resistant *Staphylococcus aureus* | (11) |
| USA300 | SMC6979 | Methicillin resistant *Staphylococcus aureus* | (12) |
|  |  |  |  |
| ***Streptococcus* spp*.*** | | | |
| *S. sanguinis* | SMC7474 | Strain SK36 | (13) |
| *S. constellatus* | SMC7155 | *Streptococcus milleri* group | (14) |
| *S. intermedius* | SMC7156 | *Streptococcus milleri* group | (14) |
| *S. anginosus* | SMC5342 | *Streptococcus milleri* group | (14) |
|  |  |  |  |
| ***Prevotella*** **spp.** |  |  |  |
| *P. melaninogenica* | SMC6965 | ATCC25845 | (15) |
| *P. intermedia* | SMC5371 | ATCC25611 | (15) |
|  |  |  |  |
| ***E. coli*** |  |  |  |
| SM10 λpir | SMC32 | Used as a conjugation partner for introducing pEX18-based plasmids. |  |
| S17 λpir | SMC117 | Used as a conjugation partner for introducing pMQ30-based plasmids. |  |
|  |  |  |  |
| **Plasmids** |  |  |  |
| pEX18Gm-Δ*lasR* | DH123 | PA14 *lasR* in-frame deletion construct; Gm^R^ | (2) |
| pMQ30 + *lasR* | DH3548 | For complementing WT *lasR* gene at the native locus; Gm^R^ | (16) |

**References**

1. L. G. Rahme *et al.*, Common virulence factors for bacterial pathogenicity in plants and animals. *Science* **268**, 1899-1902 (1995).

2. D. A. Hogan, A. Vik, R. Kolter, A *Pseudomonas aeruginosa* quorum-sensing molecule influences *Candida albicans* morphology. *Mol Microbiol* **54**, 1212-1223 (2004).

3. C. Cugini, D. K. Morales, D. A. Hogan, *Candida albicans*-produced farnesol stimulates *Pseudomonas* quinolone signal production in LasR-defective *Pseudomonas aeruginosa* strains. *Microbiology* **156**, 3096-3107 (2010).

4. C. E. Harty *et al.*, Ethanol Stimulates Trehalose Production through a SpoT-DksA-AlgU-Dependent Pathway in *Pseudomonas aeruginosa*. *J Bacteriol* **201**, e00794-00718 (2019).

5. L. E. P. Dietrich, A. Price-Whelan, A. Petersen, M. Whiteley, D. K. Newman, The phenazine pyocyanin is a terminal signalling factor in the quorum sensing network of *Pseudomonas aeruginosa*. *Mol Microbiol* **61**, 1308-1321 (2006).

6. E. E. Smith *et al.*, Genetic adaptation by *Pseudomonas aeruginosa* to the airways of cystic fibrosis patients. *Proc Natl Acad Sci USA* **103**, 8487-8492 (2006).

7. D. L. Mould, N. J. Botelho, D. A. Hogan, J. B. Goldberg, Intraspecies signaling between common variants of *Pseudomonas aeruginosa i*ncreases production of quorum-sensing-controlled virulence factors. *mBio* **11**, e01865-01820 (2020).

8. M. Whiteley, K. M. Lee, E. P. Greenberg, Identification of genes controlled by quorum sensing in *Pseudomonas aeruginosa*. *Proc Natl Acad Sci U S A* **96**, 13904-13909 (1999).

9. Q. Yu *et al.*, *In vitro* evaluation of tobramycin and aztreonam versus *Pseudomonas aeruginosa* biofilms on cystic fibrosis-derived human airway epithelial cells. *J Antimicrob Chemother* **67**, 2673-2681 (2012).

10. E. S. Duthie, Variation in the antigenic composition of staphylococcal coagulase. *J Gen Microbiol* **7**, 320-326 (1952).

11. T. R. Field, C. D. Sibley, M. D. Parkins, H. R. Rabin, M. G. Surette, The genus *Prevotella* in cystic fibrosis airways. *Anaerobe* **16**, 337-344 (2010).

12. L. K. McDougal *et al.*, Pulsed-field gel electrophoresis typing of oxacillin-resistant *Staphylococcus aureus* isolates from the United States: establishing a national database. *J Clin Microbiol* **41**, 5113-5120 (2003).

13. M. Kilian, K. Holmgren, Ecology and nature of immunoglobulin A1 protease-producing streptococci in the human oral cavity and pharynx. *Infect Immun* **31**, 868-873 (1981).

14. J. E. Scott *et al.*, *Pseudomonas aeruginosa* can inhibit growth of Streptococcal species via siderophore production. *J Bacteriol* **201**, e00014-00019 (2019).

15. H. Shah, D. M. Collins, *Prevotella*, a new genus to include *Bacteroides melaninogenicus* and related species formerly classified in the genus *Bacteroides*. *Int J Syst Bacteriol* **40**, 205-208 (1990).

16. M. E. Clay *et al.*, *Pseudomonas aeruginosa lasR* mutant fitness in microoxia is supported by an Anr-regulated oxygen-binding hemerythrin. *Proc Natl Acad Sci U S A* **117**, 3167-3173 (2020).
