# Supplementary material for: Community composition shapes microbial-specific phenotypes in a cystic fibrosis polymicrobial model system
Source: eLife. 2023 Jan 20;12:e81604. doi: 10.7554/eLife.81604 (PMC9897730; doi:10.7554/eLife.81604)
Supplement: Supplementary file 3. [file elife-81604-supp3.docx]

Supplementary File 3. Species abundances in community types used for *in silico* modeling of metabolic flux. Abundances for each species were taken from (1)

|  | *P. aeruginosa* | *S. sanguinis* | *P. melaninogenica* | *S. aureus* |
| --- | --- | --- | --- | --- |
| *Pseudomonas-*dominated (Pa.D) | 77.6% | 3.0% | 6.2% | 1.6% |
| *Streptococcus-*dominated (Strep.D) | 4.4% | 49.5% | 16.9% | 1.7% |
| Pa.M1 | 55.6% | 13.6% | 10.2% | 1.2% |
| Pa.M2 | 67.7% | 2.7% | 10.4% | 2.5% |

1. T. H. Hampton, D. Thomas, C. van der Gast, G. A. O’Toole, B. A. Stanton, Mild cystic fibrosis lung disease is associated with bacterial community stability. *Microbiol Spectr* **9**, e00029-00021 (2021).
